# Supplementary figures and images for: The African swine fever virus p22 inhibits the JAK-STAT signaling pathway by promoting the TAX1BP1-mediated degradation of the type I interferon receptor
Source: PLoS Pathog. 2025 Jul 16;21(7):e1013319. doi: 10.1371/journal.ppat.1013319 (PMC12266391; doi:10.1371/journal.ppat.1013319)

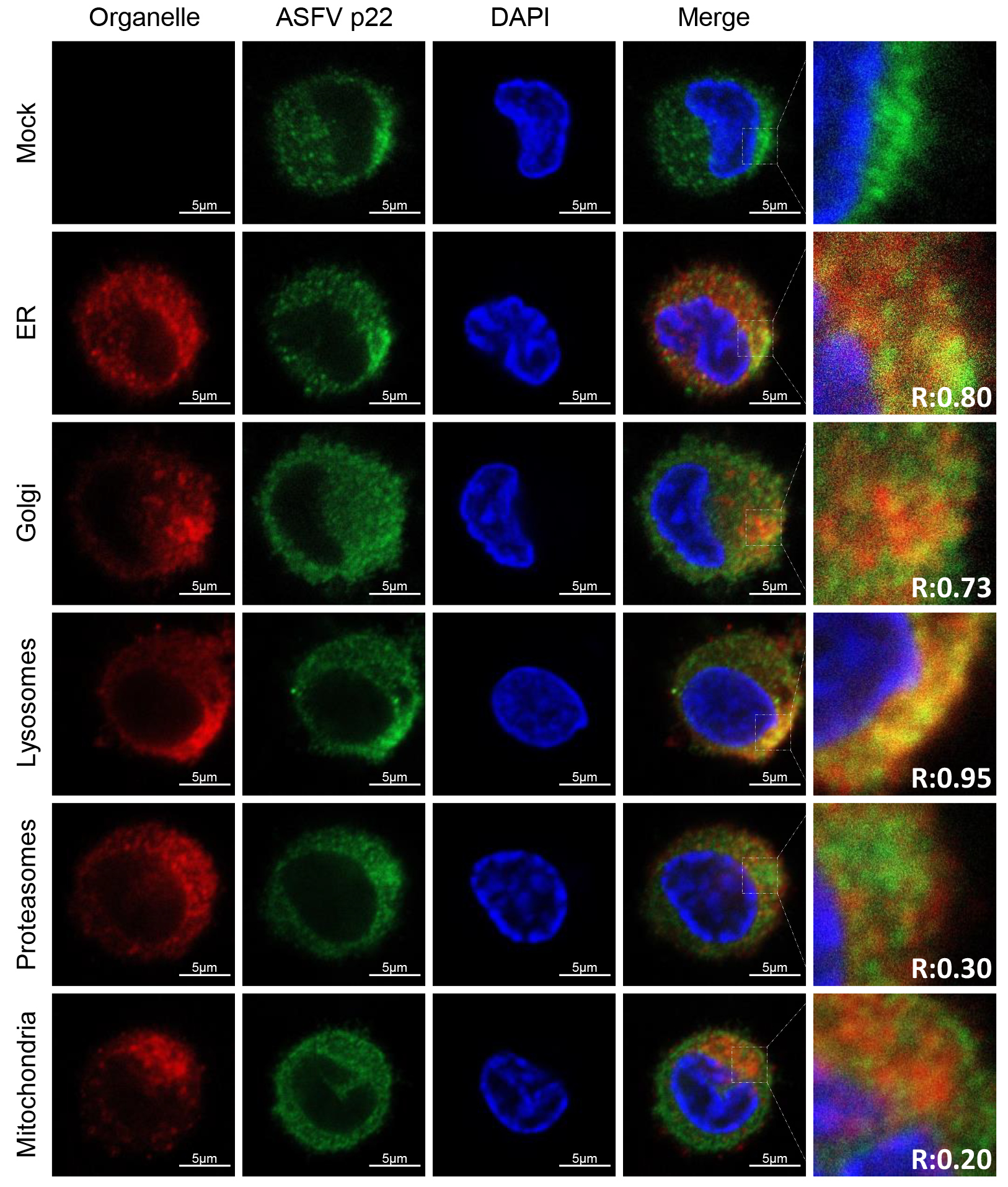

Supplement: S1 Fig — PAMs were infected with ASFV (MOI = 1). At 24 hpi, the cells were fixed with 4% paraformaldehyde. The subcellular localization of p22 was determined by immunofluorescence using the indicated antibodies, and the nuclei were stained with DAPI and subjected to confocal microscopy. The colocalization of p22 and organelle markers was analyzed using the Coloc2 tool in ImageJ, and the colocalization results represented as Pearson’s R value. Scale bar = 5 μm. (TIF) [file ppat.1013319.s001.tif]

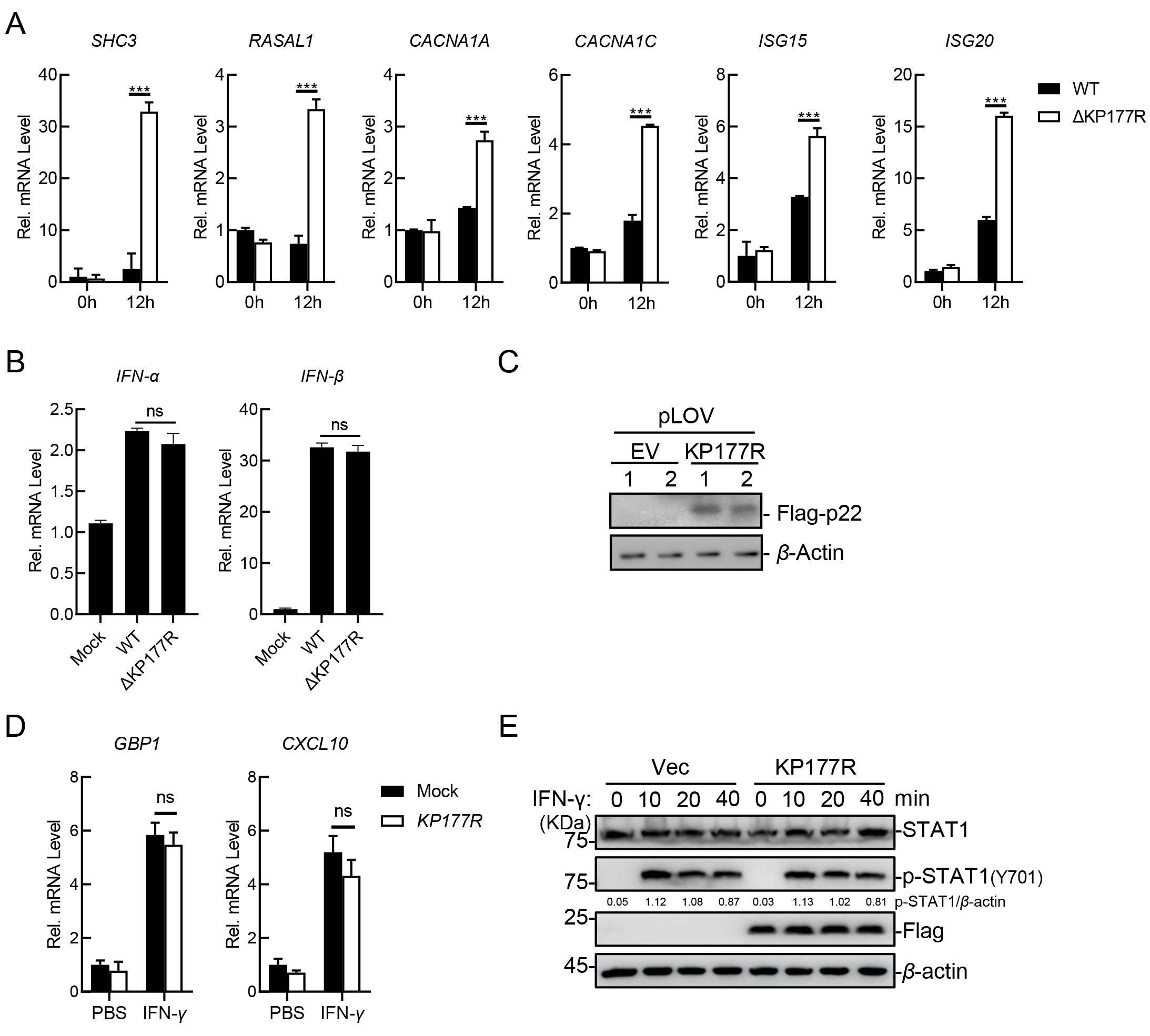

Supplement: S2 Fig — (A) PAMs were infected with ASFV-WT or ASFV-ΔKP177R (MOI = 3). At 12 hpi, the total RNA was extracted using TRIzol reagent. Subsequently, the mRNA transcription of indicated genes was examined by RT-qPCR. (B) PAMs were infected with ASFV-WT or ASFV-ΔKP177R (MOI = 1). At 24 hpi, the cells were subjected to isolation of cellular total RNAs followed by quantification of transcription level of indicated genes by RT-qPCR. (C) The stable cells overexpressing p22 or wild-type (WT) IBRS-2 cells were lysed for western blotting analysis using the indicated antibodies. (D) HEK293T cells transfected with pFlag-KP177R was treated with IFN-γ. The cells were subjected to isolation of cellular total RNAs followed by quantification of transcription level of indicated genes by RT-qPCR. (E) HEK293T cells transfected with pFlag-KP177R was treated with 20 ng/mL IFN-γ for the indicated hours. Western blotting analysis was performed using the indicated antibodies. (TIF) [file ppat.1013319.s002.tif]

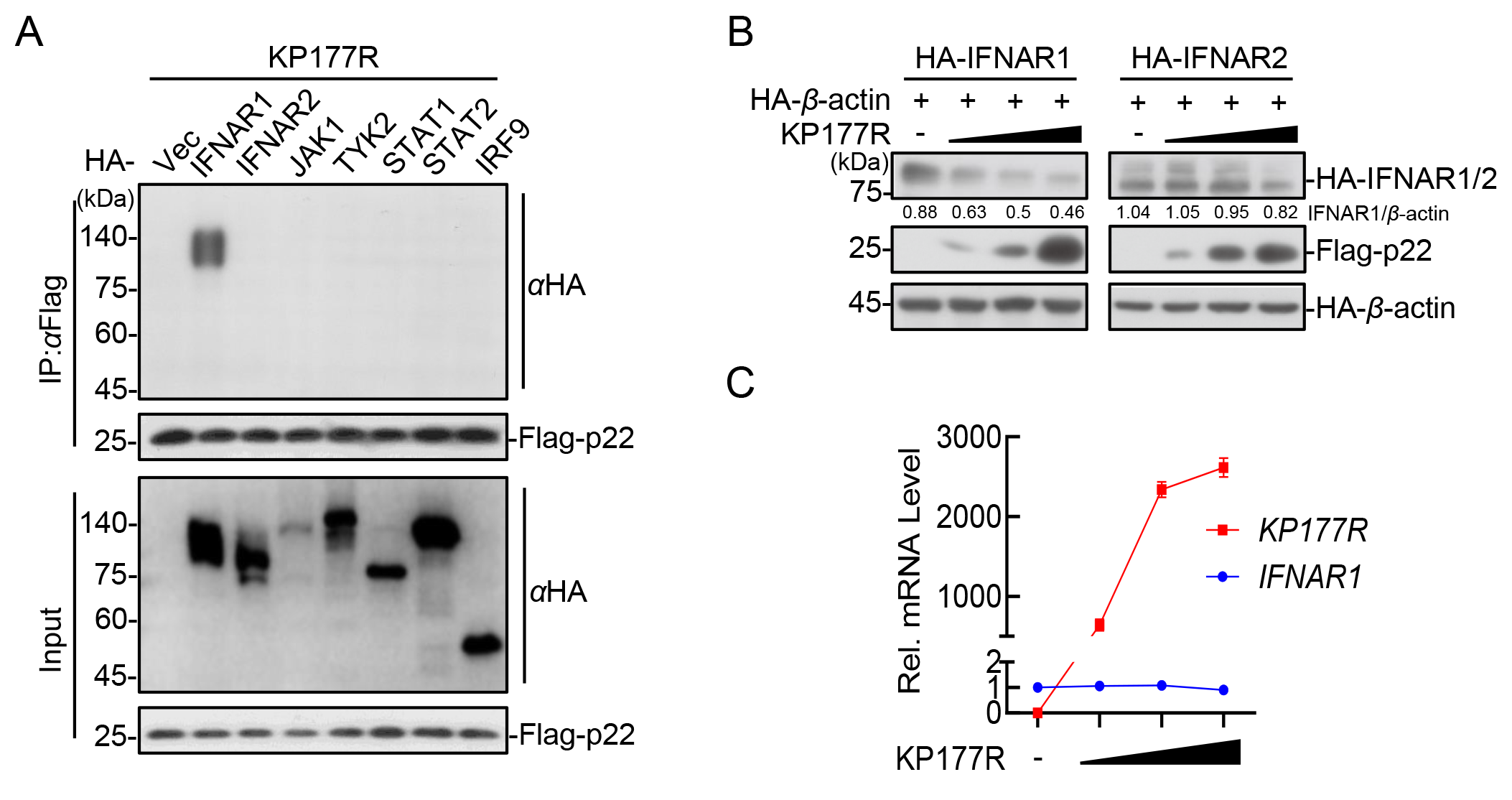

Supplement: S3 Fig — (A) HEK293T cells were cotransfected with the plasmids expressing HA-IFNAR1, -IFNAR2, -JAK1, -TYK2, -STAT1, -STAT2, or -IRF9, and Flag-p22 then lysed and analyzed using co-IP with anti-Flag MAb, followed by western blotting analysis using the indicated antibodies. (B) HEK293T cells were cotransfected with different amounts of the p22-expressing plasmid with pHA-β-actin, and pHA-IFNAR1 or pHA-IFNAR2, followed by western blotting analysis using the indicated antibodies at 24 hpt. The densitometric analysis of the protein expression levels was performed using the ImageJ software. (C) HEK293T cells were transfected with different amounts of the p22-expressing plasmid, and then the total RNA was extracted and analyzed using RT-qPCR at 24 hpt. (TIF) [file ppat.1013319.s003.tif]

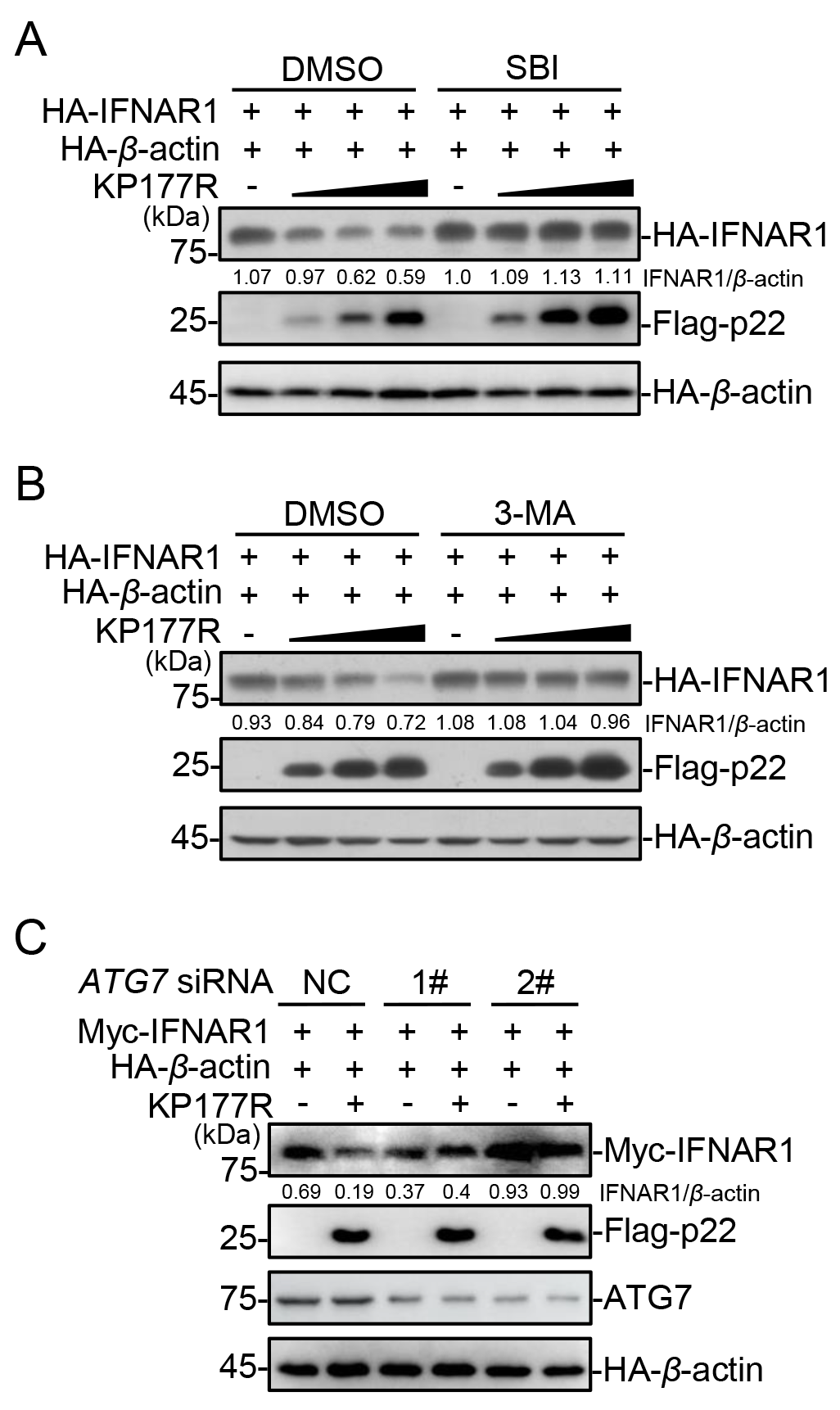

Supplement: S4 Fig — (A and B) HEK293T cells were cotransfected with different amounts of the p22-expressing plasmid with pHA-IFNAR1 and pHA-β-actin. Then, the cells were treated with DMSO, SBI, or 3-MA at 24 hpt, followed by western blotting analysis using the indicated antibodies. (C) HEK293T cells were transfected with siRNAs for 24 hours, followed by the cells were cotransfected with the plasmids pMyc-IFNAR1, pHA-β-actin, and pFlag-KP177R or pRK (Vec) and then western blotting analysis using the indicated antibodies. The densitometric analysis of the protein expression levels was performed using the ImageJ software. (TIF) [file ppat.1013319.s004.tif]

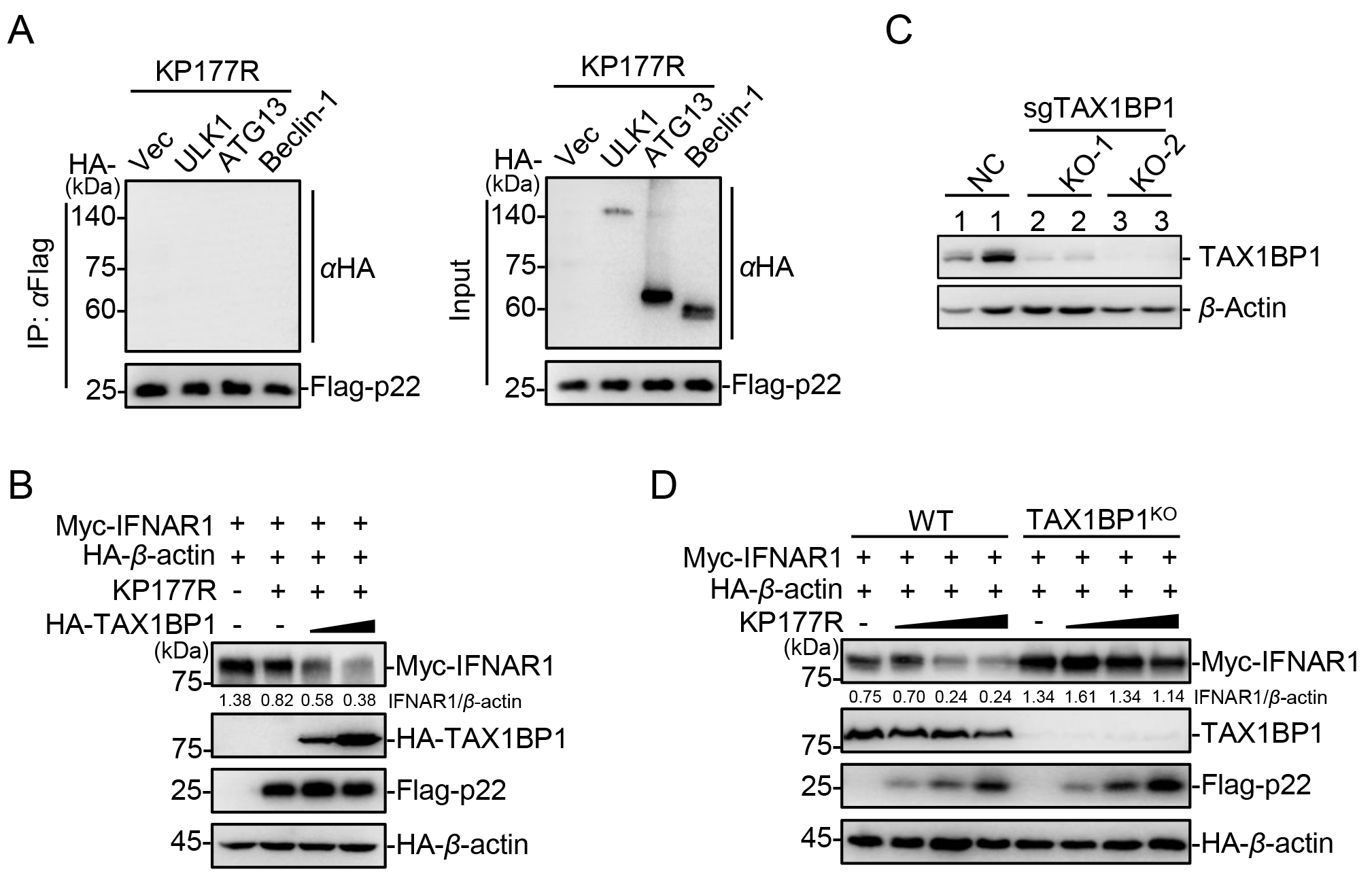

Supplement: S5 Fig — (A) HEK293T cells were cotransfected with the plasmids expressing HA-ULK1, -ATG13, or -beclin-1, and Flag-p22, and then lysed for co-IP using anti-Flag MAb, followed by western blotting analysis using the indicated antibodies. (B) HEK293T cells were transfected with different amounts of the HA-TAX1BP1-expressing plasmid with pMyc-IFNAR1, pHA-β-actin, and pFlag-KP177R, followed by western blotting analysis using the indicated antibodies at 24 hpt. (C) The TAX1BP1-knockout or wild-type (WT) HEK293T cells were lysed for western blotting analysis using the indicated antibodies. (D) The TAX1BP1-knockout or WT HEK293T cells were transfected with different amounts of the Flag-p22-expressing plasmid at 24 hpt, followed by western blotting analysis using the indicated antibodies. The densitometric analysis of the protein expression levels was performed using the ImageJ software. (TIF) [file ppat.1013319.s005.tif]

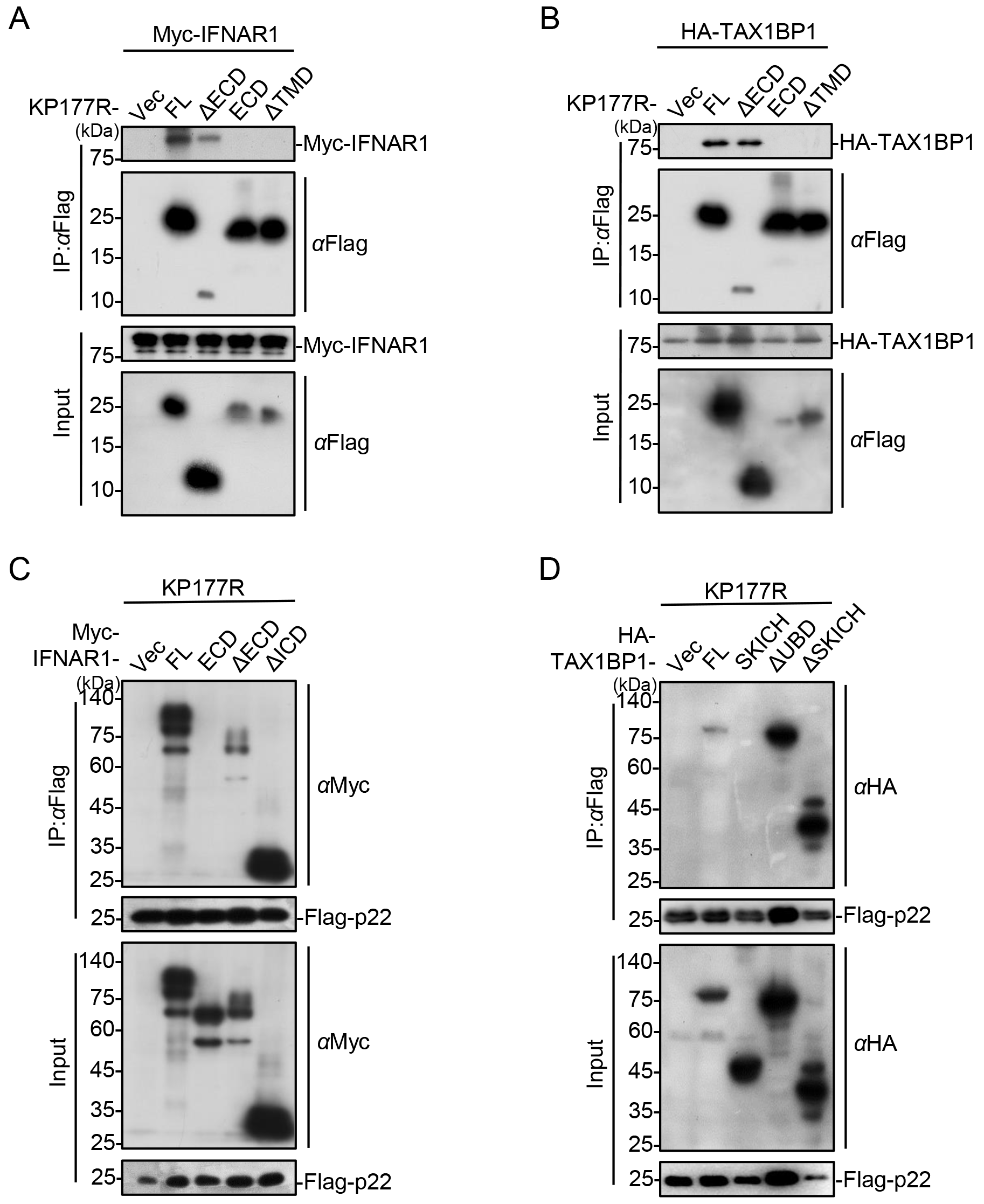

Supplement: S6 Fig — (A and B) HEK293T cells were cotransfected with the plasmids expressing Myc-IFNAR1 or HA-TAX1BP1 and Flag-p22 or the plasmids expressing the truncated Flag-p22 mutants, and then lysed for co-IP assay using anti-Flag MAb, followed by western blotting analysis using the indicated antibodies. (C) HEK293T cells were cotransfected with the plasmids expressing Flag-p22 and Myc-IFNAR1 or the plasmids expressing the truncated Myc-IFNAR1 mutants, and then lysed for co-IP assay using anti-Flag MAb, followed by western blotting analysis using the indicated antibodies. (D) HEK293T cells were cotransfected with the plasmids expressing Flag-p22 and HA-TAX1BP1 or the plasmids expressing the truncated HA-TAX1BP1 mutants and then lysed for co-IP assay using anti-Flag MAb, followed by western blotting analysis using the indicated antibodies. (TIF) [file ppat.1013319.s006.tif]
