# Supplementary material for: The African swine fever virus p22 inhibits the JAK-STAT signaling pathway by promoting the TAX1BP1-mediated degradation of the type I interferon receptor
Source: PLoS Pathog. 2025 Jul 16;21(7):e1013319. doi: 10.1371/journal.ppat.1013319 (PMC12266391; doi:10.1371/journal.ppat.1013319)
Supplement: S1 Table — (DOCX) [file ppat.1013319.s007.docx]

**S1 Table.** **Primers used in this study**

| **Primers** | **Sequences (**5′-3′**)** |
| --- | --- |
| hum-*GAPDH*-F | GACAAGCTTCCCGTTCTCAG |
| hum-*GAPDH*-R | GAGTCAACGGATTTGGTCGT |
| hum-*CCL2*-F | AGAATCACCAGCAGCAAGTGTCC |
| hum-*CCL2*-R | TCCTGAACCCACTTCTGCTTGG |
| hum-*IL-8*-F | GAGAGTGATTGAGAGTGGACCAC |
| hum-*IL-8*-R | CACAACCCTCTGCACCCAGTTT |
| hum-*TNF-α*-F | GCCGCATCGCCGTCTCCTAC |
| hum-*TNF-α*-R | CCTCAGCCCCCTCTGGGGTC |
| hum-*STAT1*-F | GTGGAAAGACAGCCCTGCAT |
| hum-*STAT1*-R | ACTGGACCCCTGTCTTCAAGAC |
| hum-*IFIT1*-F | TCATCAGGTCAAGGATAGTC |
| hum-*IFIT1*-R | CCACACTGTATTTGGTGTCTAGG |
| hum-*RSAD2*-F | GAGCGCCACAAAGAAGTGTC |
| hum-*RSAD2*-R | CAAGAAAGTTGGGTACCAATCCA |
| hum-*GBP1*-F | TAGCAGACTTCTGTTCCTACATCT |
| hum-*GBP1*-R | CCACTGCTGATGGCATTGAC GT |
| hum-*CXCL10*-F | GGTGAGAAGAGATGTCTGAATCC |
| hum-*CXCL10*-R | GTCCATCCTTGGAAGCACTGCA |
| hum-*IFNAR1*-F | GACCCTAGTGCTCGTCGC |
| hum-*IFNAR1*-R | CGACAGACTCATCGCTCCTG |
| sus-*SHC3*-F | AGGGGCTGCTGAAGAGAGAT |
| sus-*SHC3*-R | TCCTTTGTCCGGATCGTGC |
| sus-*RASAL1*-F | TGCACGTGTGAGAGACTTCC |
| sus-*RASAL1*-R | TGCGACACAGGGATAGAGGA |
| sus-*CACNA1A*-F | GTTCACTACAACCAGCCCGA |
| sus-*CACNA1A*-R | GGGTTTTATGACGGCCCAGA |
| sus-*CACNA1C*-F | CGTGCATCAGCATTGTCGAG |
| sus-*CACNA1C*-R | TCATCCTCCGGAAAGGGGAT |
| sus-*ISG15*-F | GATGCTGGGAGGCAAGGA |
| sus-*ISG15*-R | CAGGATGCTCAGTGGСTСТСТ |
| sus-*ISG20*-F | CACAGCTCGGAACATGGAGG |
| sus-*ISG20*-R | TTCCACCGAGTTGTGTCCCA |
| sus-*GAPDH*-F | ACATGGCCTCCAAGGAGTAAGA |
| sus-*GAPDH*-R | GATCGAGTTGGGGCTGTGACT |
| sus-*CCL2*-F | TGCCCAGCCAGATGCAATTA |
| sus-*CCL2*-R | TGCTGCTGGTGACTCTTCTG |
| sus-*IL-8*-F | TGGCAGTTTTCCTGCTTTCT |
| sus-*IL-8*-R | CAGTGGGGTCCACTCTCAAT |
| sus-*TNF-α*-F | GCCCAAGGACTCAGATCATC |
| sus-*TNF-α*-R | GGCATTGGCATACCCACTCT |
| sus-*STAT1*-F | CAAAGGAAGCCCCAGAACCT |
| sus-*STAT1*-R | ACTTGTTCCATGTCACGTCAA |
| sus-*IFIT1*-F | TCCGACACGCAGTCAAGTTT |
| sus-*IFIT1*-R | TGTAGCAAAGCCCTGTCTGG |
| sus-*RSAD2*-F | ATTACCACTTCACCCGCCAG |
| sus-*RSAD2*-R | TTGCTCACGATGCTGACACT |
| sus-*IFN-α*-F | GCTCCTGGCACAAATGAGGA |
| sus-*IFN-α*-R | ATGGCTTGAGCCTTCTGGAC |
| sus-*IFN-β*-F | GTTGCCTGGGACTCCTCAAT |
| sus- *IFN-β*-R | ACGGTTTCATTCCAGCCAGT |
| ASFV-*KP177R*-F | ATGTTTAATATTAAAATGACAATT |
| ASFV-*KP177R*-R | TGCATGTTTATGATTTCTAGGTAA |
| sg*TAX1BP1*-F | CACCGTGGGTTGGTATATTCAAGGT |
| sg*TAX1BP1*-R | AAACACCTTGAATATACCAACCCAC |
| si*ATG7*#1 | CAGACAAGAAGCUCCUUCUTT |
| si*ATG7*#2 | CAGCCUGGCAUUUGAUAAATT |
| si*NC* | UUCUCCGAACGUGUCACGUTT |
