# Supplementary material for: The African swine fever virus p22 inhibits the JAK-STAT signaling pathway by promoting the TAX1BP1-mediated degradation of the type I interferon receptor
Source: PLoS Pathog. 2025 Jul 16;21(7):e1013319. doi: 10.1371/journal.ppat.1013319 (PMC12266391; doi:10.1371/journal.ppat.1013319)
Supplement: S2 Table — (DOCX) [file ppat.1013319.s008.docx]

**S2 Table. Primers used for constructing the transfer vector pOK12-p72-EGFP-Δ*KP177R***

| **Primers** | **Sequences (5′-3′)** |
| --- | --- |
| KP177R-L-F | GGTACCCGGGAGCTCGAATTCCATCTATACAAGTATGATAAATCGT |
| KP177R-L-R | CATCTCTCACGAGATCGTGACATGTCTACTCCAATTTCTCTG |
| KP177R-M-F | CAGAGAAATTGGAGTAGACATGTCACGATCTCGTGAGAGATG |
| KP177R-M-R | GATACGATGAGCCTAATGTATTTATTCCTGTGAGATCATGGCAGCT |
| KP177R-R-F | AGCTGCCATGATCTCACAGGAATAAATACATTAGGCTCATCGTATC |
| KP177R-R-R | GTCTGCAGAAGCTTCGAATTCGGGAGCCAACGCTAATCGAGC |
