# Supplementary material for: The African swine fever virus p22 inhibits the JAK-STAT signaling pathway by promoting the TAX1BP1-mediated degradation of the type I interferon receptor
Source: PLoS Pathog. 2025 Jul 16;21(7):e1013319. doi: 10.1371/journal.ppat.1013319 (PMC12266391; doi:10.1371/journal.ppat.1013319)
Supplement: Attachment — Submitted filename: Response Letter-p22-R1.docx [file ppat.1013319.s010.docx]

**Point by point response letter**

**Dear Editors,**

We are grateful for the opportunity to revise and improve our manuscript. We greatly appreciate the positive comments and valuable suggestions. Based on the reviewers’ comments, we have performed several experiments and made appropriate modifications in the revised manuscript. The following is a point-by-point response to the reviewers’ comments. The changes are highlighted in blue in the revised manuscript.

**Reviewers' Comments:** Reviewer's Responses to Questions

**Part I – Summary**

Please use this section to discuss strengths/weaknesses of study, novelty/significance, general execution and scholarship.

Reviewer #1: In this study, the authors described that ASFV internal envelope membrane protein P22 degrades IFNAR1 through autophagy pathway by relying on the selective autophagy receptor TAX1BP1, and inhibits the JAK-STAT signaling pathway. These findings clarify the biological functions of p22 in ASFV replication and uncover a novel antagonistic mechanism of the virus by autophagic degradation of IFNAR1. In general, this study is well designed, clearly presented, and provides compelling insights. However, several concerns should be addressed to improve the quality of the paper.

Reviewer #2: In the manuscript by Ren et al. (PPATHOGENS-D-25-00461), the authors identified the internal envelope membrane protein (p22) of ASFV as a negative regulator of JAK-STAT signaling pathway and conducted a series of experiments to show its role on IFNAR1 stability. The authors demonstrated that p22 interacted and promoted autophagic degradation of IFNAR1 by recruiting autophagy receptor TAX1BP1 to IFNAR1. Besides, they also demonstrated that ASFV-ΔKP177R virus failed to inhibit IFN-induced *ISG* expression compared to ASFV-WT infection, suggesting critical role of ASFV structural protein p22 to evade host immune response. Together this work convincingly showed that p22 regulates JAK-STAT signaling through autophagic degradation of IFNAR1.

Reviewer #3: Large knowledge gaps regarding the biological characteristics of African swine fever virus (ASFV) structural proteins have severely hindered the development of vaccines against African swine fever (ASF). In this study, Ren et al. investigated the functional role of viral structural protein p22 involved in the immune evasion of ASFV, and revealed that p22 inhibited the JAK-STAT signaling by promoting the TAX1BP1-mediated degradation of the type I interferon receptor. This study provides convincing evidence that the selective autophagy receptor TAX1BP1 is involved in interaction with p22 and IFNAR1 through biochemical and cell-based assays. Furthermore, the p22 was shown to promote the association of the TAX1BP1 with the IFNAR1 and facilitate the autophagic degradation of IFNAR1 to inhibit innate immunity. These findings are of significant interest to researchers in the fields of autophagy and virology. However, the following comments should be addressed to further refine the manuscript.

**Part II – Major Issues: Key Experiments Required for Acceptance**

Please use this section to detail the key new experiments or modifications of existing experiments that should be absolutely required to validate study conclusions.

Generally, there should be no more than 3 such required experiments or major modifications for a "Major Revision" recommendation. If more than 3 experiments are necessary to validate the study conclusions, then you are encouraged to recommend "Reject".

**Reviewer #1:**

1. Fig. 1C showed that p22 was at a low expression level at 24 hpi, while the RNA-seq data authors compared the difference between ASFV-ΔKP177R and ASFV-WT at 12 and 18 hpi, was the conclusion reached by the authors due to p22?

**Reply:** Thank you for your valuable comments. p22 is a structural component of ASFV and localized on the internal envelope membrane of virion, which enables it to antagonize the host antiviral immune response upon viral infection. Meanwhile, to confirm the deletion of the *KP177R* gene, PAMs were infected with the ASFV-ΔKP177R or ASFV-WT at a multiplicity of infection (MOI) of 0.1, and the infection duration was extended to 72 hours. As shown in Fig. 1C, the expression of p22 was detectable from 24 hours to 72 hours post ASFV-WT infection, but not in the ASFV-ΔKP177R-infected PAMs. Whereas, during assays concerning the RNA-seq data presented in Fig. 2A, PAMs were infected with ASFV-WT at an MOI of 3, and the higher MOI of infected viruses lead to increased expression of p22. Thus, the cells were harvested at 12 and 18 hpi. To avoid any potential misinterpretation by readers, the detailed MOI of all assays was clearly indicated in the revised manuscript (lines 183, 606, 612, 975, 983, 984, 986, 993-995, 998, 1001, 1027, 1041, 1059, 1082, 1136, 1145, 1147 and Supporting information lines 13, 22, 24).

**2**. The effects of p22 on the expression of STAT1, STAT2, p-STAT1 and p-STAT2 were detected (Fig. 2E, Fig. 3C, Fig. 8D). However, the expression of these proteins in the control group should be consistent, the authors should check these WBs.

**Reply:** Thanks for valuable suggestions. The phosphorylation of STAT1 and STAT2 is the hallmark of the activation of the JAK-STAT signaling pathway, and the expression of STAT1, STAT2, p-STAT1 and p-STAT2 were detectable upon viral infection or p22-expressing plasmid transfection.

In these experiments, the housekeeping protein *β*-actin served as sample loading control, while viral protein p30 was used for identifying viral infections. No significant differences were observed in the controls among different groups. When assessing the changes in p-STAT1 and p-STAT2, the protein levels of p-STAT1 and p-STAT2 were normalized to their corresponding *β*-actin. Our results showed that, IFN-*β* treatment significantly triggered the phosphorylation of STAT1/2, and the expression of STAT1/2 was also detectable in Fig. 2E, Fig. 3C, Fig. 8D. Whereas, various cells were used in Fig. 2E (PAMs), Fig. 3C (HEK293T cells and IBRS-2 cells), and Fig. 8D (IBRS-2 cells). The protein abundance, including but not limited to STAT1/2, varies across diverse cell types, namely the cell-type-specific expression.

**3**. In Fig. 3D and Fig. 8C, the fold-change of *STAT1*, *IFIT1*, and *RSAD2* after treatment by IFN-β in IBRS-2 cells was not consistent, the stimulation difference should be described.

**Reply:** We greatly appreciate the reviewer’s professional comments. In Fig. 3D and Fig. 8C, IFN-*β* treatment (20 ng/mL) induced the transcription of *STAT1, IFIT1,* and *RSAD2* genes in IBRS-2 cells*.* However, different passages of IBRS-2 cells exhibit varying sensitivity to identical interferon stimulation. To avoid potential misinterpretations, replicate experiments were conducted, and the fold-change of *ISGs* with comparable activation levels upon IFN-*β* treatment were included in the updated Fig. 8C.

**4**. The LC3 lipidation experiments presented in Figure 5C and 5E should be repeated to enhance clarity, and the analyses of protein intensity should be included in the figure.

Reply: We thank the reviewer for these constructive suggestions. As suggested, these experiments have been repeated, and the figures have been updated as Fig. 5C and 5E.

**5**. In Fig. 5D, it showed that p22 promoted the co-localization of TAX1BP1 and LC3, and there were three colors in the figure. How did the author perform Pearson's R value calculation should be described.

**Reply:** Pearson's R value was calculated to quantify the degree of colocalization between blue-labeled TAX1BP1 and green-labeled LC3, and the detailed description were added in the results section (lines 299, 300).

6. p22 induces autophagy in cells, why LC3 aggregates into the nucleus after addition of p22?

**Reply:** Thank you for this thoughtful comment. The assay has been reperformed in IBRS-2 cells. As shown in the updated Fig. 5D, in the absence of p22, LC3 was diffusely distributed in IBRS-2 cells. And the presence of p22 promoted the formation of EGFP-LC3 spots mainly in the cytoplasm.

**7**. In Fig. 5B, the expression of IFNAR1 was particularly up-regulated in the CQ treatment group?

**Reply:** Thanks for your valuable comments. It has been reported that intracellular protein kinase p38 and casein kinase 1*α* facilitate the phosphorylation of IFNAR1 through their kinase activity, which subsequently triggers the ubiquitination of IFNAR1 by E3 ubiquitin ligase *β*-TRCP, leading to its degradation via the lysosomal pathway (1, 2, 3). Consistently, CQ treatment led to a substantial increase of the endogenous IFNAR1 with or without p22 expression in Fig. 5B.

1. Bhattacharya S, Qian J, Tzimas C, Baker DP, Koumenis C, Diehl JA, et al. Role of p38 protein kinase in the ligand-independent ubiquitination and down-regulation of the IFNAR1 chain of type I interferon receptor. The Journal of biological chemistry. 2011; 286(25):22069-22076.

2. Liu J, Carvalho LP, Bhattacharya S, Carbone CJ, Kumar KG, Leu NA, et al. Mammalian casein kinase 1alpha and its leishmanial ortholog regulate stability of IFNAR1 and type I interferon signaling. Molecular and cellular biology. 2009; 29(24):6401-6412.

3. Guo T, Zuo Y, Qian L, Liu J, Yuan Y, Xu K, Miao Y, Feng Q, Chen X, Jin L, Zhang L, Dong C, Xiong S, Zheng H. ADP-ribosyltransferase PARP11 modulates the interferon antiviral response by mono-ADP-ribosylating the ubiquitin E3 ligase *β*-TrCP. Nat Microbiol. 2019; 4(11):1872-1884.

**8**. Fig. 8 The author wants to prove that the KP177R-TMD domain is its key functional domain. Why KP177R-ΔECD (including ICD and TMD) is chosen instead of KP177R-ΔTMD for the experiment?

**Reply**: To investigate the function of KP177R-TMD, the effects of various mutants on the activation of STAT1 promoter and the degradation of IFNAR1 were examined. The mutant KP177R-ΔECD, including ICD and TMD, was sufficient to remarkably inhibit the IFN-*β*-triggered activation of the STAT1 promoter and promote the degradation of IFNAR1, whereas mutants KP177R-ECD and KP177R-ΔTMD with deletion of the TMD had no influence on the activation of the IFN response. These findings suggest that the TMD of ASFV p22 is essential for its function. As you have astutely observed, the mutant KP177R-ΔTMD, which losses the effect on the JAK-STAT signaling pathway activation, can also be used for verification. And we have strategically employed target-specific positive mutant (KP177R-ΔECD) throughout the validation procedures as shown in Fig. 8C and D.

**9**. A previous study demonstrated that TAX1BP1 was downregulated and cleaved by Coxsackievirus B3 (PMID: 34149637). The temporal expression profile of endogenous TAX1BP1 following ASFV infection should be systematically examined. Furthermore, this study should also rule out the potential effects of ASFV proteases on TAX1BP1 expression.

**Reply**: We thank the reviewer for this constructive suggestion. As suggested, the temporal expression profile of endogenous TAX1BP1 following ASFV infection were examined. Our results showed that both ASFV-WT and ASFV-△KP177R infection downregulated the expression of TAX1BP1, which suggested that ASFV encodes one or more proteins that regulate its expression through either direct or indirect mechanisms. Further in-depth investigations concerning this issue will be of great interest.

**Reviewer #2**:

**1**. The authors performed RNA-seq in PAM cells infected with ASFV-WT and ASFV-ΔKR177R virus and found that JAK-STAT signaling pathway is enriched by comparison of WT and ΔKR177R virus infection. The authors performed qPCR to validate the negative role of p22 in JAK-STAT signaling pathway, while the *ISGs* validated such as *IFIT1*, *STAT1*, *RSAD2* is not the most upregulated genes from RNA-seq. Again, the JAK-STAT is not the top enriched pathways from the KEGG analysis. Validation of the top enriched pathways and genes are required to justify the functional significance of p22 depletion on ASFV induced immune response.

**Reply**: Thank you for your valuable and thoughtful comments. Firstly, to verify the credibility of our RNA-seq data, six DEGs of the top enriched pathways (*SHC3* and *RASAL1* from the RAS signaling pathway, *CACNA1A* and *CACNA1C* from the MAPK signaling pathway, *ISG15* and *ISG20* from the JAK-STAT signaling pathway) were selected for transcription level validation via RT-qPCR assay. Our results suggested that the mRNA transcription of these genes was significantly upregulated in the ASFV-ΔKP177R infection group comparing with the ASFV-WT infection group, thereby corroborating the reliability of the RNA-seq data (updated Fig. S2A, lines 189-199, 1144-1147 and Supporting information lines 21-24). Besides, as known, ASFV strains including SY18, HLJ/18 and BA71V are highly sensitive to IFNs-I treatment, and the ability of ASFV to counteract with the JAK-STAT signaling pathway exhibited intimate connection between the pathogenicity of ASFV. Thus, we focused on the JAK-STAT signaling pathway, and the most ubiquitously monitored hallmarks *IFIT1, STAT1,* and *RSAD2* were selected as a surrogate marker to delineate *ISG* dynamics. Further investigations concerning the functional significance of p22 depletion on the other signaling pathways including RAS or MAPK signaling are necessary and of great significance.

**2**. In their previous study, the authors demonstrated that IFN-*γ* signaling is critical during ASFV infection in PAM cells. Does p22 of ASFV play any role in IFN-*γ* triggered signaling pathway?

**Reply**: We thank the reviewer for this constructive suggestion. To investigate whether p22 plays a role in IFN-*γ*-triggered signaling pathways, the transcription level of *GBP1* and *CXCL10,* and the phosphorylation of STAT1 were measured in the p22-overexpressing HEK293T cells upon IFN-*γ* (20 ng/mL) treatment. The results showed that p22 had no effect on the IFN-*γ*-triggered transcription of *GBP1* and *CXCL10*, and the phosphorylation of STAT1 (updated Fig. S2D and S2E, lines 243-248, 1151-1156 and Supporting information lines 28-33).

**Reviewer #3:**

**1**. The subcellular localization of p22 was analyzed in the cells with ectopic expression, and it is essential to examine the subcellular localization of p22 in ASFV-infected cells.

**Reply**: We greatly appreciate the reviewer’s professional comments. The subcellular localization of p22 in ASFV-infected cells were examined. Consistently, p22 was present in the cytoplasm, exhibited a perinuclear distribution, and localized in the endoplasmic reticulum (ER), Golgi apparatus, and lysosomes in the ASFV-infected PAMs. However, its presence was negligible in the mitochondria and proteasomes (updated Fig. S1, lines 156, 543-548, 1135-1138 and Supporting information lines 12-15).

**2**. The authors should discuss which proteins, among pS273R or pMGF360-9L, pH240R, pMGF505-7R, and pF778R, primarily regulate the JAK-STAT signaling.

**Reply**: Thank you for this thoughtful suggestion, and this issue has been discussed in this new version (lines 469-475). As a giant and complex DNA virus, ASFV encodes several proteins to counteract host IFN response, such as pS273R、pMGF360-9L、pH240R、pMGF505-7R and pF778R. These proteins exhibit varying levels of abundance across different stages of viral replication and interact with distinct molecular targets within the JAK-STAT pathway during viral infection. It is speculated that they orchestrate the modulation of host inflammation via finely-tuned cooperative mechanisms.

**Part III – Minor Issues: Editorial and Data Presentation Modifications**

Please use this section for editorial suggestions as well as relatively minor modifications of existing data that would enhance clarity.

**Reviewer #1:**

**1**. Introduction section lacks detailing knowledge on ASFV and autophagy, as well as related studies that explore the interaction between individual ASFV proteins and autophagy (PMID: 37566637, PMID: 37442088, PMID: 33830435).

**Reply**: Thank you for your suggestion. To make our manuscript reflecting the state of this field more precisely, we have added several key references, and further reviewed the introduction in the new version (lines 82-90).

**2**. *KP177R* has previously been demonstrated to be non-essential (PMID: 34073222), which should also be included in the Introduction rather than the Discussion section.

**Reply**: Corrected as suggested (lines 134-135).

**3**. If the *KP177R* deletion virus induces the upregulation of *ISGs*, could this imply that the virus is also inducing IFN? Alternatively, might the observed differences be due to the presence of type I IFNs in the supernatant of the KP177R-deleted virus preparations, which are absent in the wild-type virus?

**Reply**: Thank you for this thoughtful comment. The transcription levels of type I IFNs in the ASFV-WT or ASFV-ΔKP177R infected PAMs were measured as suggested. The results showed that the deletion of the *KP177R* gene did not affect the transcription level of *IFN-α* and *IFN-β* (updated Fig. S2B, lines 207-212, 1147-1149, and Supporting information lines 24-26). Meanwhile, cells were treated with exogenous IFN-*β* at a concentration significantly higher than these produced during viral infection.

**4**. TAX1BP1-ΔUBD already exists in Fig. 7C, but its schematic diagram is shown in Fig. 8A.

**Reply**: Thank you for your kindly suggestion. We have checked and refined the details in this section (lines 371, 372, 374).

**5**. In Fig. S5A and B, the expression of KP177R-ΔECD in input group was high, but it was low in IP group?

**Reply**: Thank you for your suggestion. This phenomenon was consistently observed across multiple repeated experiments, which may stem from that Myc-IFNAR1 or Myc-TAX1BP1 and the anti-Flag magnetic beads competitively bind to KP177R-ΔECD-Flag, thereby reducing its enrichment efficiency. Despite the lower enrichment of KP177R-ΔECD, interactions between KP177R-ΔECD-Flag and both IFNAR1 and TAX1BP1 were still detectable, demonstrating that p22 binds to IFNAR1 and TAX1BP1 *via* its transmembrane domain.

**6**. The format writing of the expression of p22 (KP177R) should be consistent throughout the text.

**Reply**: Corrected as suggested (lines 175, 1025, 1145, 1087, 1160, 1194, 1195, and Supporting information line 37, 72, 73).

**Reviewer #2:**

**1**. The plasmids used in this study should clarify the sources. For example, pSTAT1-Fluc, it's originally from commercial resources or generated in the lab.

**Reply**: We thank the reviewer for this detailed suggestion. The plasmids pNF-*κ*B-Fluc, pSTAT1-Fluc, pRL-TK, psPAX2, and pMD2.0G, and the pLOV vector were kindly provided by Prof. Hong-Bing Shu at Wuhan University. Among them, pSTAT1-Fluc and pRL-TK were originally purchased from QIAGEN, and pNF-*κ*B-Fluc was originally provided by Professor Gary Johnson at North Carolina State University, USA. As suggested, the detailed information has been supplemented in the revised manuscript (lines 575-577).

**2**. The author demonstrated that p22 reduced the phosphorylation levels of STAT1 and STAT2. Please clarify the phosphorylation types and residues of STATs, such as tyrosine phosphorylation (pY701), serine phosphorylation (pS727) of STAT1 or other type of phosphorylation antibody used in this study.

**Reply**: Corrected as suggested. Tyrosine phosphorylation of STAT1 at tyrosine 701, and tyrosine phosphorylation of STAT2 at tyrosine 690 were detected using indicated antibodies.

**3**. In line 207, STATs are transcription factors, not adaptor proteins.

**Reply**: Corrected as suggested.

**4**. In line 240, two “that” in the text.

**Reply**: Corrected as suggested.

**Reviewer #3:**

**1**. The IBRS-2 cell lines stably expressing p22 and TAX1BP1 knockdown cell lines were constructed in this study, it is essential to provide evidence in the Supporting Information confirming the successful establishment and validation of these stable cell lines.

**Reply**: Thank you for your valuable comments. As suggested, the expression level of p22 and TAX1BP1 in indicated cell lines were confirmed *via* western blotting analysis. The results showed that p22 was detectable, and TAX1BP1 was efficiently knocked down especially in KO-2 cell line (updated Fig. S2C and 5C, lines 232, 343-345, 1149-1151, 1185-1187 and Supporting information lines 26-28, 62-64).

**2**. Fig. 1F, A more detailed description of the assay and result is required to enhance the clarity.

**Reply**: Corrected as suggested. More detailed descriptions have been included in the Results, Materials, and Methods sections of Fig. 1F (lines 169-173, 607-609).

**3**. The manuscript fails to adequately introduce the broader literature and understanding of virus interaction with autophagy. Several studies have shown that RNA viruses, including enteroviruses (doi：10.1080/15548627.2022.2153572), Coronavirus (doi：10.1080/15548627.2020.1817280) , Influenza A viruses (doi: 10.1080/15548627.2022.2162798), Picornavirus (doi：10.1080/15548627.2024.2350270), subvert the host autophagy pathway at multiple stages. Furthermore, there exist a substantial amount of literature on viral manipulation of selective autophagy receptors, including p62, T6BP, NDP52, and NBR1, which have not been discussed in this context.

**Reply**: We thank the reviewer for this constructive suggestion, and relevant literatures have been summarized and incorporated into the Introduction section (lines 77-81, 96-98).

**4**. Fig. 8C: There is a notable discrepancy between Figs. 8B and 8C regarding the JAK-STAT signaling of the ΔECD mutant. In Fig. 8B, the ΔECD mutant exhibited significantly higher inhibition of the signaling compared to the FL (full length) version, however, when examining their effects on *ISGs* expression, no significant difference is observed between the FL and the ΔECD mutant. The authors should provide an explanation for this observation in the Discussion section.

**Reply**: Thanks for this helpful suggestion. Through multiple independent replicate experiments, we concluded that both the FL and the ΔECD mutant of p22 robustly antagonized the JAK-STAT signaling. While inter-experimental variability in the extent of suppression between the FL and the ΔECD mutant of p22 was observed (potentially attributable to fluctuations in transfection efficiency across biological replicates), thus we selected representative datasets that demonstrate comparable inhibitory effects for inclusion in the revised Fig. 8B. Critically, this methodological adjustment ensures the preservation of the scientific integrity of our original conclusions.
